# Psychoeducational Challenges in Spanish Children With Dyslexia and Their Parents’ Stress During the COVID-19 Pandemic

**DOI:** 10.3389/fpsyg.2021.648000

**Published:** 2021-05-28

**Authors:** Manuel Soriano-Ferrer, Manuel Ramón Morte-Soriano, John Begeny, Elisa Piedra-Martínez

**Affiliations:** ^1^Department of Developmental and Educational Psychology, University of Valencia, Valencia, Spain; ^2^Department of Psychology, North Carolina State University, Raleigh, NC, United States; ^3^Department of Special Education, University of Azuay, Cuenca, Ecuador

**Keywords:** dyslexia, COVID-19, psychoeducational impact, parent stress, quarantine

## Abstract

**Background:**

Research during 2020 has been rapidly attending to the impact of COVID-19 on various dimensions of wellbeing (e.g., physical, psychological, lifestyle and routines) on adults and children around the world. However, less attention has focused on the psychoeducational impact on children and their families. To our knowledge, no currently available studies have looked specifically at the impact of COVID-19 on students with dyslexia and their families. Research on this topic is needed to offer greater support for this population of students and their families.

**Objective:**

The main objective of this paper is to examine the psychoeducational impact of the required COVID-19 quarantine in Spain among children with dyslexia and their families.

**Method:**

A sample of 32 children with dyslexia and their mothers participated in this study.

**Measures:**

Children and adolescents with dyslexia and their mother completed several measures before the required national quarantine in Spain and again during the quarantine. Children completed measures of depression, state anxiety, reading activity, and reading motivation. Mothers provided demographic information and completed measures related to students’ emotional and behavioral difficulties as well as parenting stress, parental distress, and a questionnaire about educational problems during quarantine.

**Results:**

Major findings showed that during quarantine, children with dyslexia had increased levels of depression and anxiety symptoms, and parents perceived their children as having more emotional symptoms, hyperactivity-inattention, and conduct problems. During quarantine, children and adolescents with dyslexia also showed less reading activity and less reading motivation. Parents also reported significantly more stress, during quarantine compared to pre-quarantine conditions. Some demographic and psychological variables predicted children’s state anxiety as well parental stress. The questionnaire related to impacts of quarantine also revealed several important findings. For example, nearly all parents of children with dyslexia reported (a) difficulties in establishing study routines, (b) that the quarantine negatively affected their child’s learning, and (c) that they did not receive sufficient help from teachers on how to support their child’s learning. Additionally, the vast majority of the parents were very worried about the child’s learning and school success, the child’s motivation and interest in reading, the child’s peer relations, and the professional skills of the child’s teacher.

**Conclusion:**

This study offers a preliminary investigation into this topic and elucidates several psychoeducational challenges that children with dyslexia and their families have experienced during the quarantine in Spain. Study findings highlight the need to provide immediate support for children with dyslexia and emphasizes the importance of developing prevention programs to mitigate any future negative impacts of COVID-19 on children with dyslexia and their parents.

## Introduction

Dyslexia is a neurodevelopmental disorder of neurological origin and characterized by impaired reading acquisition despite the presence of adequate intelligence, education, and socioeconomic background to learn to read (American Psychiatric Association [APA], 2014; Adrián-Ventura et al., 2020). Dyslexia impacts approximately 6% of elementary and secondary students in Spain (Jiménez et al., 2009; González et al., 2010) and an estimated 5–15% of the global population (American Psychiatric Association [APA], 2014). The first decades of research in dyslexia were mainly devoted to the cognitive and biological underpinning of the disorder (Vellutino et al., 2004; Soriano-Ferrer and Piedra-Martínez, 2017). Compared to students with average to above average reading skills, accumulated evidence suggests that students with dyslexia can show a range of reading-related (e.g., pseudoword reading, spelling, vocabulary) and cognitive (e.g., rapid naming, verbal, working memory) difficulties (Soriano-Ferrer and Miranda, 2010; Kudo et al., 2015; Soriano-Ferrer et al., 2016; Morte-Soriano et al., 2020).

In recent years, a growing body of evidence suggests that children and adolescents with dyslexia also show increased risk of experiencing mental health difficulties (Swanson and Vaughn, 2016; Heiman and Olenik-Shemesh, 2020). The repeated experiences of failure faced by students with dyslexia appear to increase their vulnerability to experiencing internalized disorders (e.g., anxiety and depression) and lower opinions of their academic competence. Studies examining psychological wellbeing for those with a learning disability (LD) offer relevant data because dyslexia is the most common LD. For instance, the meta-analysis by Nelson and Harwood (2011b) revealed that around 70% of students with a LD experience greater anxiety symptoms than students without a LD, and there were no differences between self-reports and parent reports of anxiety. Also, a study by Panicker and Chelliah (2016) revealed that a relatively higher percentage of students with a specific LD had severe stress (16%), severe anxiety (23%), and/or severe depression (14%). Nelson and Harwood (2011a) also completed a meta-analysis to understand depression among students with a LD and found higher scores on measures of depression than students’ peers without a LD, which was true regardless of who reported depression-related symptoms (i.e., self-report, parent, or teacher). Similarly, the meta-analysis by Francis et al. (2019) found that poor readers were at moderate risk (at statistically significant levels) for experiencing anxiety and depression, and Mammarella et al. (2016) found that students with dyslexia have worse symptoms of depression than individuals with non-verbal learning disabilities.

Some studies (e.g., Lee and Zentall, 2012; Soriano-Ferrer et al., 2014; Wolters et al., 2014; Soriano-Ferrer and Morte-Soriano, 2017) have also looked specifically at the psychoeducational wellbeing of children with dyslexia and found that this population of students have more negative self-perceptions as readers (e.g., feeling less competent in reading, having more difficulty, and not liking reading), have lower motivation to read (e.g., they more commonly avoid reading activities) as assessed by self-reports or by teachers’ reports, and demonstrate lower utility of reading. Thus, students with dyslexia read less for enjoyment and engage less in reading activities. Lee and Zentall (2017) also found that students with dyslexia maintained low reading motivation and decreased their reading for school as they transitioned to middle school.

Dyslexia presents significant challenges for the student and it can also have negative impacts on their parents (Delany, 2017). Mothers, in particular, show higher levels of stress and depression and report significant impacts on family and increased difficulties in everyday life (Bonifacci et al., 2014). Snowling et al. (2007) observed that approximately 74% of parents reported that their child’s difficulties had a mild to severe impact on family life and that mothers of children with dyslexia had higher levels of stress and depression. Using the Parental Stress Index (PSI), results of Bonifacci et al. (2014) seem to suggest that the effect on the family system of having a child with dyslexia may be specific to parent-child interactions and on worries regarding the needs of the child with a LD. Also, Carotenuto et al. (2017) found that mothers of children affected by dyslexia reported higher rates of parental stress and difficulty on the PSI according to indexes of Parental Distress, Parent-child Dysfunctional Interaction, and “Difficult Child” (which measures a parent’s perceptions of the child’s behavior with respect to behaviors that often make parenting easier or more difficult.

### COVID-19 and the Impact of National Quarantines on Children and Their Families

Because of the significant and widespread health threat of the coronavirus (COVID-19) pandemic, many governments worldwide decreed home-based quarantines as an approach to contain the expansion of COVID-19. For example, Spain was in state of alarm from March 14, 2020, until June 21, 2020, with few exceptions (e.g., to purchase essential items such as food, attend health centers, or take a walk close to home). According to the United Nations Educational, Scientific, and Cultural Organization [UNESCO], 2020), school closures caused by the coronavirus pandemic have already affected over 1.5 billion students and their families. The disruption of in-person learning is unprecedented and, as was highlighted in the report of Di Pietro et al. (2020), the move to remote learning environments in numerous countries could have significant and negative impacts on children’s academic learning as well as their cognitive and non-cognitive skills.

School closures–and therefore remote learning–creates restricted mobility and social isolation for students and the overall set of circumstances presents major challenges for children and their families’ psychological wellbeing (Cachón-Zagalaz et al., 2020; Griffith, 2020; Mazza et al., 2020a,b). Several recent studies have used parent reports about the impact of quarantine on childrens’ or adolescents’ mental health. For instance, with a sample from Spain, Erades and Morales (2020) found that 70% of parents reported their children as having at least one negative emotional reaction during the pandemic (e.g., 31% were reported to have sleep problems and 24% had behavioral problems). Gómez-Becerra et al. (2020) indicated that the highest psychological difficulties for Spanish children and adolescents appear to be connected to emotional symptoms, behavioral problems, and overall difficulty. The authors also reported that as the quarantine continues over time, the presence of psychological difficulties generally increase. Within western countries, emotional and behavioral changes in their children were observed by parents in the first weeks of lockdown (Francisco et al., 2020; Orgilés et al., 2020). For example, in the study by Orgilés et al. (2020), 86% of parents from Spain perceived changes in their children’s emotional state and behavior during the quarantine (e.g., 77% had difficulty concentrating, 52% experienced significant boredom, 39% experienced irritability, 38% showed restlessness, and 38% had notable nervousness). In another study carried out 7 weeks after lockdown and utilizing parent report, Orgilés et al. (2021) found that 56% of children scored above the clinical cut-off point for anxiety and 26.4% scored above the clinical cut-off for depression.

A few studies over the past months have used self-report measures for children and adolescents. Xie et al. (2020) found higher levels of depression among study participants in China and Duan et al. (2020) found that 23% of respondents from China suffered from depressive symptoms. Cao et al. (2020) similarly reported higher levels of generalized anxiety in college students from China (0.9% severe, 2.7% moderate, and 21.3% mild anxiety) during the quarantine. Duan et al. (2020) also reported (though not with exact percentages) higher levels of anxiety, with gender differences (i.e., more anxiety in females than in males) and age differences (i.e., more anxiety in adolescents than in children). Moreover, 55% and 36% reported that the pandemic has affected their learning or ability to graduate, respectively. Overall, in the relatively short duration during which researchers have examined impacts of COVID-19, most studies have reported negative psychological effects on children and adolescents who are impacted by national quarantines, and these impacts are occurring across numerous countries around the world (Brooks et al., 2020; Francisco et al., 2020; Liang et al., 2020; Orgilés et al., 2020, 2021).

At the family level, parents are usually the ones who must help their child(ren) to cope with the additional stress and emotional difficulties (e.g., anxiety and symptoms of depression) caused by school closures, remote learning, and children’s confinement at home (Brown et al., 2020; Fegert et al., 2020; Halvorsen et al., 2020; Spinelli et al., 2020). Brooks et al. (2020) reviewed 24 studies published between 2004 and 2019 in different countries about the psychological impact of quarantine on adults and concluded that most studies reported negative psychological effects, including post-traumatic stress symptoms, confusion, and anger. The studies reviewed by Brooks et al. (2020) also suggested that longer durations of quarantine generally correspond with worse mental health symptoms. In a study of 3,055 adults from Spain aged 18–88 years, Rodríguez-Rey et al. (2020) concluded that 37% of these participants showed psychological distress due to the COVID-19 pandemic. Likewise, in China 40.4% of youth were prone to psychological distress (Liang et al., 2020).

Indeed, for the vast majority of individuals around the world, the life condition of families suddenly and deeply changed as a result of the COVID-19 pandemic. As one example, during quarantine in the home environment, the educational role of parents has become even much crucial than before. Children often have only their parents to provide support with homework (when necessary) and parents can become the central figure needed to promote a positive learning and developmental experience (Wang et al., 2020). Furthermore, parents of children with pre-existing psychological and behavioral difficulties are reporting problems with managing their children at home over this extended period (Colizzi et al., 2020; Marchetti et al., 2020a). Therefore, dealing with confinement and trying to balance factors such as personal life, professional responsibilities, and the education of one’s child(ren) can be a stressful experience (Marchetti et al., 2020a,b; Mazza et al., 2020a,b; Spinelli et al., 2020). The study by Spinelli et al. (2020) in Italy is the only published report to date that explored the effect of COVID-19 on parents’ stress. Their results showed that COVID-19 puts parents of children aged 2–14 years old at a higher risk of experiencing individual and dyadic distress during quarantine, potentially impairing their ability to be supportive caregivers. The researchers suggested that the lack of support many children are able to receive in such a difficult moment may be a reason for their more pronounced psychological symptoms. Furthermore, some studies have shown that prolonged exposure to parental stress can result in another important phenomenon occurring during the pandemic: the so-called *parental related exhaustion*. Parental related exhaustion has been defined as feelings of being overextended and depleted of one’s emotional and physical resources (Mikolajczak et al., 2018), which in turn influence psychological well-being of children. Indeed, in their study on parental-related exhaustion during the COVID-19 pandemic, Marchetti et al. (2020b) found that more than 80% of their sample reported high levels of distress and 17% experienced significant parenting-related exhaustion.

Some previous studies (e.g., Cao et al., 2020; Duan et al., 2020; Gómez-Becerra et al., 2020; Orgilés et al., 2020, 2021) have linked mental health problems, both in children and adults, with variables related to COVID-19 (e.g., fear of illness, death, having a family/friend infected,…), while other studies (e.g., Mikolajczak et al., 2018; Colizzi et al., 2020; Liang et al., 2020; Marchetti et al., 2020a,b; Spinelli et al., 2020) have related them to socio-demographic characteristics (e.g., having a larger number of children, lacking a domestic partner) or child features (e.g., special needs, externalizing symptoms, ADHD, behavior problems). However, further studies should examine this issue in even greater depth.

### Purpose of the Present Study and Research Questions

Despite the important and relatively rapid development of research and reporting on psychological and educational impacts of national quarantine policies resulting from COVID-19, research in this area is still emerging and several gaps exist. For example, research focusing on psychological impacts of children aged approximately 9–14 years is limited and we are not aware of a single study that has specifically evaluated children with dyslexia. Given the research summarized at the beginning of this report, research must begin to carefully examine the impacts of school closures on students with dyslexia because they are already vulnerable to both learning and psychological difficulties. Additionally, there are no known psychoeducational-focused studies that have explored the impacts of COVID-19 quarantines on both children and parents within the same household. Such research will help to develop a more comprehensive picture of quarantine practices and policies on specific sub-populations of individuals. Furthermore, although some studies have been able to report on individuals’ *physical* health before and during quarantine (e.g., through assessments of obesity and diabetes), we are not aware of any studies that have been able to directly assess children’s or parents’ psychological wellbeing at a time period just before quarantine and again within 1–2 months after quarantine began. Such data offer more direct, rather than retrospective, accounts of wellbeing and are therefore methodologically stronger in design.

Collectively, there are good reasons to expect that school closures together with self-isolation and social distancing present particular challenges for children and adolescents with dyslexia and we may expect that quarantines resulting from COVID-19 could increase psychological and educational problems in both parents and their children with dyslexia. However, this remains an empirical question and the main objective of this study is to examine the psychological and educational impact of COVID-19 quarantine on students with dyslexia (e.g., depression, anxiety, reading motivation, reading habits) and their parents (e.g., distress). In doing so, we seek to address some existing limitations within the rapidly emerging COVID-19 research on psychological wellbeing and aim to answer the following research questions (RQs)–based on both pre-quarantine and concurrent-quarantine data–from a sample of Spanish children with dyslexia and their parents.

RQ1: Based on self-report, what are the psychological (e.g., symptoms of depression and state anxiety) and reading-related impacts (e.g., perceived value of reading and actual engagement with reading, reading habits) of the 2020 national quarantine on children with dyslexia?

RQ2: For children with dyslexia, how does the quarantine impact the child’s emotional and behavioral (e.g., conduct problems, hyperactivity-inattention problems) state as reported by a parent?

RQ3: To what extent does quarantine impact parental stress and other aspects of mental health for parents who have a child with dyslexia?

RQ4: Are there any pre-quarantine predictors of negative impact on children’s emotional wellbeing or parent distress during the quarantine?

RQ5: During quarantine for children with dyslexia, what are some key educational conditions and impacts, as reported by a parent?

## Materials and Methods

### Participants

Participants included 32 children with dyslexia as well as the mother of each child participant. Like each mother, fathers of the child participants were also asked to participate by completing measures used in the study; however, as will be described later, in only a small number of instances did the father collaboratively complete a measure with the child participant’s mother. For brevity, we refer to the school-aged participants as “child” or “children” even though some participants’ ages could be classified as early adolescence. Additional details about all study participants are provided below in the respective sub-sections.

The study respected all principles outlined in the current legislation on research investigations and was approved by the Research Ethics Committee of the University of Valencia, which is regulated by Ethical Principles for Medical Research Involving Human Subjects and aligns with the Declaration of Helsinki (World Medical Association [WMA], 2013). For example, prior to commencing any study procedures, parents’ informed consent was obtained.

#### Children With Dyslexia

All 32 child participants were Caucasian, lived in Spain at the time of the study, and spoke Spanish as their primary language. Moreover, children were attending 21 schools (8 primary schools and 13 secondary schools). They ranged from 9 to 14 years old (mean age = 10 years and 11 months, *SD* = 1 year and 5 months. Seventeen identified as male and fifteen as female).

These participants were recruited from referrals to a multi-year investigation that began several years before the COVID-19 pandemic. Preliminary selection of potentially eligible child participants was consistent with past studies (e.g., Pereira-Laird et al., 1999; Soriano-Ferrer and Miranda, 2010; Begeny et al., 2011; Soriano-Ferrer et al., 2011), such that potential dyslexia participants in the present study were recommended by their teachers based on students’ low reading achievement in the classroom. The actual presence of dyslexia for child participants was then determined by using the DSM-5 (American Psychiatric Association [APA], 2014) criteria specified for identifying students with a specific LD: (a) scores of 80 or above on an intelligence test (Cattell and Cattell, 2006), in order to exclude students with cognitive impairments; (b) no evidence or history of neurological damage, significant economic disadvantage, emotional disturbance, hearing or vision abnormalities, or any other major handicapping condition; and (c) a reading achievement score at or below the 25th percentile on the word-reading and/or pseudoword-reading skill subtests (accuracy and/or speed) from the Standardized Reading Skills Battery (PROLEC-R, Cuetos et al., 2002; PROLEC-SE, Ramos and Cuetos, 2003).

Three participants (9.4%) had been retained in a grade, 46.9% had received services in the school’s resource room prior to this study, and 87.5% were reported by the teacher as needing help with homework. Eleven (34.4%) of the children had no siblings, 53.1% had a sibling, and 12.5% had two or more siblings. Table 1 shows the characteristics of all child participants.

**TABLE 1:** Characteristics of child participants.

	*M*	*SD*
Age	10.96	1.49
IQ	106.78	10.64
Word reading accuracy	34.16	5.32
Word reading speed	79.60	32.28
Word reading fluency	49.95	19.64
Pseudoword reading accuracy	28.84	7.26
Pseudoword reading speed	95.69	33.28
Pseudoword reading fluency	34.03	15.07
**Sex**	***N***	%
Male	17	53.1
Female	15	46.9
***Academic Qs***	***N***	%
Grade retention	3	9.4
Resource room	15	46.9
Homework Help	28	87.7
***Siblings***	***N***	%
No siblings	11	34.4%
1 Sibling	17	53.1%
>2	4	12.5%

#### Parents/Caregivers of Children With Dyslexia

Each mother of the 32 aforementioned children participated in this study. Like the child participants, these caregivers (henceforth referred to as *parents*) had also participated in past components of this multi-year investigation. Table 2 includes parents’ demographic information and includes fathers’ information to provide a clearer picture of child participants’ parents. Mothers’ age ranged from 30 to 52 years (mean age = 42 years, 1 month, *SD* = 4 years, 1 month), and fathers’ age ranged from 38 to 60 years (mean age = 44 years, 2 months, *SD* = 4 years, 8 months). Most parents’ highest level of education (53.1% of mothers and 65.5% of fathers) was a diploma from Secondary School. Approximately 22–24% of mothers and fathers had a degree from a university. All fathers and 69% of mothers reported having a paid job. In accordance with the International Standard Classification of Occupations (International Labour Organization [ILO], 2012), the occupations of fathers and mothers were distributed into occupations with four levels of skills (see Table 2). Due to the COVID-19 pandemic, some parent participants lost their jobs (9.4% of mothers and 3.4% of fathers), and others had their working hours reduced (18.7% of mothers and 34.5% of fathers). Additionally, 68.7% (*n* = 22) of the parents were married or living together, 9.4% (*n* = 3) reported being single or widowed, and 21.9% were separated (*n* = 7).

**TABLE 2:** Characteristics of parent participants.

	*Mothers (N* = *32)*		*Fathers (N* = *29)*	
	*M*	*SD*	*M*	*SD*
Age (years)	42.2	4.2	44.3	4.9
**Highest level of education completed**	***N***	%	***N***	%
Elementary	8	25	3	10.3
Secondary	17	53.1	19	65.5
University	7	21.9	7	24.1
***Occupation*^*a*^**	***N***	%	***N***	%
Without a paid job	10	31.2	0	0
Skill level 1	12	37.5	4	13.8
Skill level 2	5	15.6	20	69.0
Skill level 3	5	15.6	5	17.2
Skill level 4	0	0	0	0

*^*a*^Based on the International Standard Classification of Occupations (International Labour Organization [ILO], 2012).*

### Measures

Twelve total measures were used for purposes of this study, most of which were administered both before and during Spain’s 2020 national quarantine. For clarity in our method, each section specifies if the respective measure was completed before quarantine, during quarantine, or both.

#### Measures Completed by Each Child Participant

##### Factor “g” intelligence test (scale 2, form A)

This intelligence test (Cattell and Cattell, 2006) was administered to child participants prior to the quarantine to assess general mental capacity without the interference of verbal stimuli. The reliability of the Spanish adaptation of this intelligence test is 0.86. In the current sample, the Cronbach’s alpha was 0.79.

##### Standardized reading skills battery

Prior to the quarantine, each child’s word and pseudoword reading skills were measured with the Standardized Reading Skills Battery (PROLEC-R, Cuetos et al., 2002; PROLEC-SE, Ramos and Cuetos, 2003). Word reading requires the correct identification of 40 words that vary greatly in frequency, length and spelling structure (CCV, CVV, CVC, CCVC, CVVC, and VC, where C = consonant and V = vowel), and pseudoword reading consists of pronouncing 40 pseudowords, constructed by changing or adding one or two letters to each of the 40 words on the reading test. In word and pseudoword reading, task completion is timed. Two combined scores of fluency are obtained, one based on word reading fluency and the other on pseudoword reading fluency. In both cases, the child’s score consists of an accuracy score divided by the reading speed, measured as the time taken to complete the task, and then multiplied by 100. These reading batteries have adequate internal consistency with a Cronbach’s alpha >0.70 for words and pseudowords reading in both the normalization sample and the current one.

##### Children’s depression inventory-short form

Before and during quarantine, depressive symptoms were measured using the Children’s Depression Inventory-Short Form (CDI-S) (Kovacs, 1992), which is a self-assessment screening tool of 10 items. Children mark one of three statements best describing their feelings within the past 2 weeks. Items statements indicate the severity of depressive symptomatology and are arranged in either increasing or decreasing order. Total scores range from 0 to 20, whereas high sum scores indicate a more severe symptomatology. The Spanish adaptation of this inventory for children, ranging from 9 to 15 years old, has adequate reliability with a Cronbach’s alpha of 0.71 (Del Barrio et al., 2002). In the current sample, the Cronbach’s alpha was 0.69.

##### State-trait anxiety inventory for children

Before and during quarantine, children completed the state anxiety subscale of the Spanish adaptation of the State-Trait Anxiety Inventory for Children (STAIC) (Spielberger, 2014). The state anxiety subscale evaluates the current state of anxiety, asking how respondents feel “right now,” using items that measure subjective feelings of apprehension, tension, nervousness, worry, and activation/arousal of the autonomic nervous system. This subscale includes 20 Likert-type items, scoring on a three-point scale, ranging from 1 (almost never) to 3 (almost always). The Cronbach’s alpha is reported to be 0.94 (Spielberger, 2014). In the current sample, the Cronbach’s alpha was 0.90.

##### Motivation to read profile-revised

Before and during quarantine, children responded to the Motivation to Read Profile-Revised (MRP) (Malloy et al., 2013), The 20-item survey assesses two specific dimensions of reading motivation: self-concept as a reader and value of reading. The items that focus on self-concept as a reader are designed to elicit information about students’ self-perceived competence in reading and their self-perceived perception relative to peers. The value of reading items elicits information about the value students place on reading tasks, particularly in terms of frequency of reading engagement and reading-related activities. Students respond to items using a 1–4 Likert scale, with four representing the most positive response. Cronbach’s alpha revealed a moderately high reliability for both subscales (self-concept = 81; value = 0.87) and for the full scale 0.87 (Malloy et al., 2013). In the current sample, the Cronbach’s alpha was 0.87, 0.95, and 0.97, for self-concept, value and total scale respectively.

##### Task-value scale for children

Before and during quarantine, children’s task motivation was measured in an interview using the Task-value Scale for Children (TVS-C) (Nurmi and Aunola, 2005; Aunola et al., 2006). This scale measures children’s task motivation in school and in home. In the present study, we used the three items measuring children’s intrinsic value (e.g., “How much do you like doing reading-related tasks at home?”. Each item was rated on a five-point Likert scale (1 = I do not like it at all/I dislike doing those tasks; 5 = I like it very much/I really enjoy doing those tasks). A sum score for subject-related intrinsic value was created by calculating the mean of the three scores for the three items. Cronbach’s alpha is reported to be 0.88 (Aunola et al., 2006). In the current sample, the Cronbach’s alpha was 0.84.

##### Reading activity inventory

Before and during quarantine, the Reading Activity Inventory (RAI) was used to measure the breadth (genres read) and frequency (how often a genre was read) of students’ reading (Guthrie et al., 1994). The shortened version of 10 items by Wigfield and Guthrie (1997) was used in this study. The children were asked to answer five questions about what they read (mystery books, sports books, adventure books, comic/magazine and nature books) and how often they read for fun. If the child indicated that a given kind of book was read in the last week, the child was then asked to give its title. The child then is asked to indicate how often he or she reads that kind of book, responding on a 1–4 scale from almost never to almost every day. Although there is no traditional reliability for this measure, the fall and spring administrations of the measure correlated 0.54 (*p* < 0.001), suggesting a substantial level of stability in the measure (Wigfield and Guthrie, 1997). In the current sample, the Cronbach’s alpha was 0.84 for breadth and 0.70 for frequency of reading.

#### Measures Completed by a Parent of Each Child Participant

##### Demographic information questionnaire

At the very beginning of the study, parents supplied information about their sex, level of education, occupation (based on the International Standard Classification of Occupations), and marital status. They also responded to items about the number of children being raised in the child participant’s home and the child participant’s past and current schooling (e.g., number of grade retentions, whether they received homework help).

##### Strengths and difficulties questionnaire

Before and during the quarantine, parents were asked to complete the Spanish version of the Strengths and Difficulties Questionnaire (SDQ) (Goodman, 2001; Ortuño-Sierra et al., 2017) which is designed to gather information about emotional and behavioral difficulties experienced by children from 4 to 17 years old. The current study focuses specifically on the following subscales: *emotional symptoms* (e.g., often unhappy, down-hearted), *conduct problems* (e.g., often has temper tantrums or hot temper) and *hyperactivity-inattention* (e.g., restless, overactive, cannot stay still for long). Each subscale is measured by five items, rated by a three-point scale ranging from 0 (strongly disagree) and 2 (strongly agree). To obtain the total scores, items are summed. Normative data of Español-Martín et al. (2020) that set at 90th percentile as the clinical cut-off range were used. The test has been shown to have criterion validity and good test–retest reliability after 4 and 6 months (mean = 0.62). Furthermore, the internal consistency is satisfactory, with a Cronbach’s alpha ranging from 0.57 to 0.88 (Goodman, 2001). The Spanish adaptation by Ortuño-Sierra et al. (2017) showed an internal consistency for Total Difficulties score of 0.84, ranging from 0.75 to 0.78 for the SDQ subscales. In the current sample, the Cronbach’s alpha was 0.96 for emotional problems, 0.92 for conduct problems, and 0.82 for hyperactivity-inattention.

##### Parenting stress index-short form

Before and during the quarantine, the Parenting Stress Index–Short Form (PSI-SF) was used to evaluate parenting and family characteristics–with the aim of identifying potential for parental behavior problems and child adjustment difficulties within the family system (Abidin, 1995). In this study, we used the Spanish PSI-SF (Díaz-Herrero et al., 2010, 2011). PSI-SF includes 36 items and yields a Total Stress Score from three scales (12 items per scale): Parental Distress (PD), Parent–Child Dysfunctional Interaction (PCDI), and Difficult Child (DC). Each item is scored on a five-point Likert scale, from 1 (strongly disagree) to 5 (strongly agree), with higher scores indicating greater levels of parental stress. The PD domain measures the distress experienced by parents due to personal factors, such as depression or conflict with a partner, or life restriction due to the demands of childrearing in their role as parents. The PCDI domain focuses on the perception of the child as not responsive to parental expectations. The DC subscale represents behaviors that children often engage in that may make parenting easier or more difficult. The scale also provides a measure of total stress by adding up the scores of the 36 total items. The PSI-SF shows high internal consistency (Cronbach’s alpha): 0.84 (PCDI), 0.82 (PD/DC), and 0.90 for Total Stress. In the current sample, the Cronbach’s alpha was 0.70 for PD, 0.80 for PCDI, 0.73 for DC, and 0.86 for total stress.

##### The general health questionnaire

During the quarantine, parent mental health was measured using the 12-item General Health Questionnaire (GHQ-12) (Goldberg et al., 1997), which is a self-assessment screening tool. The Spanish version of this questionnaire has good psychometric properties and the Cronbach’s alpha coefficient was 0.86 for persons of ≤65 years and 0.90 for ≥65 years (Rocha et al., 2011). The questionnaire includes response options that range from 0 (never) to 3 (usually). Total scores range from 0 to 36 with higher scores indicating higher degrees of disturbance of the general health status. A total score higher than 12 points (Percentile score >75) indicates a “tendency toward psychological problems.” In the current sample, the Cronbach’s alpha was 0.75.

##### Questionnaire about educational conditions and potential impacts of quarantine on children with dyslexia

Developed for the purposes of this study and to align specifically with educational issues potentially occurring during the COVID-19 pandemic, we used a 16-item questionnaire during the quarantine to ask parent participants questions about remote learning, time spent on learning, difficulties in homework, help from teachers, and some educational worries of parents during the national quarantine. For brevity, we will henceforth refer to this measure as the *Quarantine Questionnaire about Education* (QQE). Table 6 summarizes all questions that were asked in the QQE.

### Procedure

Parents provided informed consent during the initial evaluation of each child’s Factor “g” Intelligence Test, Standardized Reading Skills Battery, and demographic information questionnaire which occurred between November 1, 2019 and January 23, 2019. An experienced and licensed clinical psychologist (an author of this paper) administered the intelligence and reading assessments in random order and in a setting free from noise and distractions. Test sessions varied in length depending on the participant being assessed. Following these assessments, between February 2 and 11, 2020, the additional child measures were administered by the same clinical psychologist in random order through an interview. Mothers completed the measurements while their son was being interviewed. In May 2020, each evaluation administered during the quarantine was carried out in two or three sessions by means of telephone interviews or by videoconference (with Blackboard Collaborate) with the children and their parents. Thus, CDI-S, STAIC, MRP, TVR-C, RAI, SDQ, and PSI-SF were filled before and after quarantine in a random order, while GHQ-12 and QQE were filled only during confinement. This assessment corresponded to approximately 1.5 months (i.e., 45–47 days) after the national quarantine began in Spain. Parent participants who had multiple children in the household were specifically asked to respond to all questions and surveys for the specific child participant in the study. Measures intended for parents (i.e., the SDQ, PSI-SF, GHQ-12, and QQE) were mainly completed only by the child participant’s mother (81.2%; *n* = 26); however, for 18.7% (*n* = 6) of the child participants, the SDQ was completed collaboratively by both parents. The PSI-SF, GHQ-12, and QQE were completed only by mothers.

### Data Analysis

We analyzed data using SPSS version 24. First, descriptive statistics were used to describe the participants’ characteristics before quarantine (see Tables 1, 2) and to analyze educational data regarding to our RQ 5. Thus, descriptive statistics were provided as a mean and SD for continuous variables; frequencies and percentages were used for categorical variables. Regarding analysis for our RQs 1, 2, and 3 we used paired *t* tests to evaluate the significance of changes (before and during quarantine) in children with dyslexia and their parents. To control for multiple comparisons, the level of significance was established after applying the Bonferroni correction (0.05/15 = 0.003). We report effect sizes using the Cohen’s *d*, with values between 0.00 and 0.20 considered small effect sizes, values between 0.20 and 0.50 considered medium, and values above 0.80 considered large effect sizes. Finally, in order to analyze data regarding RQ 4, based on previous studies (Mikolajczak et al., 2018; Colizzi et al., 2020; Marchetti et al., 2020a,b; Spinelli et al., 2020) we carried out different multiple linear regression analyses to find out if any pre-quarantine variables influenced children’s emotional problems (e.g., anxiety) or parents’ stress during quarantine.

## Results

### Psychological and Reading-Related Impacts of Quarantine as Reported by Children With Dyslexia (RQ 1)

As measured by the CDI-S and STAIC respectively, children with dyslexia showed significantly higher levels of depression (*t*_(31)_ = −8.1, *p* = 0.001, *d* = −1.4) and state anxiety (*t*_(31)_ = −6.6, *p* = 0.001, *d* = −1.2) during the quarantine compared to before the quarantine period, with large effects sizes in both cases (see Table 3). Note that *t*-scores and Cohen’s *d* are negative when a variable increased during quarantine and are positive when is the score is lower during quarantine.

**TABLE 3:** Psychological and motivational impact of Spain’s National Quarantine on children with dyslexia.

	Before quarantine		During quarantine		*t*_(31)_	*p*	*d*
Measure	Mean	SD	Mean	SD			
Depression (CDI-S)	3.0	2.5	5.3	1.9	−8.1	0.001	−1.4
State anxiety (STAIC)	42.7	7.6	47.5	6.56	−6.6	0.001	−1.2
**Reading motivation (MRP)**							
Reading Self-Concept Subscale	21.9	3.3	21.6	2.8	1.7	0.096	−0.3
Value of Reading Subscale	25.1	4.3	22.7	5.2	6.9	0.001	1.2
Total Score on MRP	47.0	5.5	44.3	6.1	7.7	0.001	1.4
Task-Value (TVS-C)	9.1	2.7	5.9	2.1	10.1	0.001	1.8
**Reading activity (RAI)**							
Breadth of Reading	1.5	1.0	0.7	0.9	6.6	0.001	1.2
Frequency of Reading	1.1	0.4	1.0	0	1.6	0.129	0.3

**t*-scores and Cohen’s *d* are negative when a variable increased during quarantine, and are positive when is the score is lower during quarantine.*

As measured by the MRP, children with dyslexia also had significantly lower scores during the quarantine on the Value of Reading subscale (*t*_(31)_ = 6.9, *p* = 0.001, *d* = 1.2) and Reading Motivation total score (*t*_(31)_ = 7.7, *p* = 0.001, *d* = 1.4). Similarly, children’s task-value motivation, as measured by the TVS-C, also decreased at significant levels during quarantine (*t*_(31)_ = 10.1, *p* = 0.001, *d* = 1.8). With each of these changes, we observed large effect sizes (*d* range = 1.2–1.8).

Regarding reading activity, children on average reported through the RAI that they significantly decreased their breadth of reading during quarantine (*t*_(31)_ = 6.6, *p* = 0.001, *d* = 1.2) and most children decreased the types of books they read. In the before quarantine period, 18.8% (*n* = 6) of child participants indicated they did not read any type of book last week, and 81.3% (*n* = 26) read between one and three types of books. In contrast, during the quarantine, 50% (*n* = 16) reported they did not read any books last week. However, there were no statistically significant differences in children’s reported frequency of reading. Specifically, before the quarantine, 90.6% of the children with dyslexia reported they “almost never” read for fun and then 100% reported this during the quarantine. The restricted variability may have influenced a lack of statistically significant findings (i.e., even before quarantine, 90% of the children almost never read for fun).

### Parent Perspectives of Emotional and Behavioral States for Children With Dyslexia (RQ 2)

Overall, parents reported some significant changes in children’s emotional and behavioral symptoms (rated using the SDQ scale) before and during the quarantine (see Table 4). According to parents’ ratings, children with dyslexia scored significantly higher during the quarantine than before the quarantine on all three subscales: Emotional Symptoms (*t*_(31)_ = −4.5, *p* = 0.001, *d* = −0.8), Conduct problems (*t*_(31)_ = −5.1, *p* = 0.001, *d* = −0.9), and Hyperactivity-Inattention (*t*_(31)_ = −7.0, *p* = 0.001, *d* = −1.2), with large effect sizes in all cases. Table 4 also shows that the percentage of participants rated above the clinically significant range on the SDQ was higher during the quarantine period than before the quarantine. Before the quarantine, the percentage of children with clinical scores ranged from 3.1% for Hyperactivity-Inattention to 15.5% for Emotional Symptoms; however, during the quarantine, the percentage of children rated with clinically significant scores ranged from 18.8% (for Hyperactivity-Inattention) to 53.1% (for Emotional Symptoms.

**TABLE 4:** Parents’ perceived impact of Spain’s National Quarantine on emotional and behavioral difficulties of children with dyslexia.

	Before quarantine			During quarantine			*t*_(31)_	*p*	*d*
*SDQ domains*	Mean	SD	% (*N*)^*a*^	Mean	SD	% (*N*)^*a*^			
Emotional symptoms	3.1	2.5	15.6 (5)	3.9	2.5	53.1 (17)	−4.5	0.001	−0.80
Conduct problems	1.3	1.5	9.4 (3)	2.1	1.8	21.9 (7)	−5.1	0.001	−0.90
Hyperactivity-inattention	3.3	1.4	3.1 (1)	4.7	1.3	18.8 (6)	−7.0	0.001	−1.24

*^*a*^Percentage (%) and *N* with Clinically Elevated Score; *t*-scores and Cohen’s *d* are negative when a variable increased during quarantine, and are positive when is the score is lower during quarantine.*

### Mental Health Impacts of Quarantine on the Parents Who Have a Child With Dyslexia (RQ 3)

Using the PSI-SF, parent participants reported significant changes in parenting stress during the quarantine (see Table 5). For example, during quarantine parents reported higher scores on the subscale areas of Parental Distress (*t*_(31)_ = −14.4, *p* = 0.001, *d* = −2.4), Parent-Child Dysfunctional Interaction (*t*_(31)_ = −10.2, *p* = 0.001, *d* = −2.0), and Difficult Child (*t*_(31)_ = −7.2, *p* = 0.001, *d* = −1.7). Given the subscale increases in stress during the quarantine, the Total Stress scale was also significantly higher (*t*_(31)_ = −21.4, *p* = 0.001, *d* = −3.8). Furthermore, 31.3% of mothers scored above the clinically significant range (with a Percentile Score >75) on the GHQ-12, showing a tendency toward psychological difficulties during the quarantine.

**TABLE 5:** Impact of Spain’s National Quarantine on the stress of parents with a child who has dyslexia.

	Before quarantine		During quarantine		*t*_(31)_	*p*	*d*
PSI-SF	Mean	SD	Mean	SD			
PD	27.6	6.1	44.5	5.7	−14.4	0.001	−2.4
PCDI	24.0	3.3	36.5	7.2	−10.2	0.001	−2.0
DC	26.6	4.9	35.6	6.0	−7.2	0.001	−1.7
**Total stress**	**78.1**	**7.4**	**116.6**	**11.8**	−**21.4**	**0.001**	−**3.8**

*PD = Parental Distress; PCDI = Parent–Child Dysfunctional Interaction; DC = Difficult Child.*t*-scores and Cohen’s *d* are negative when a variable increased during quarantine, and are positive when is the score is lower during quarantine.*

### Predictors of Negative Impacts of Quarantine on Children’s Emotional Difficulties and Parents’ Stress (RQ 4)

Finally, in order to determine if any pre-quarantine variables influenced children’s emotional problems (e.g., anxiety) or parents’ stress during quarantine different models of multiple linear regression have been run. In the case of children, we used different pre-quarantine variables as independent variables (e.g., marital status, number of siblings, parent-child interaction,…) and level of anxiety and depression during quarantine as dependent variables. In the case of the parents, different pre-quarantine variables (e.g., marital status, number of children, child’s behavioral problems,…) were used as independent variables and the total stress score during quarantine was the dependent variable.

When predicting children’s emotional problems during confinement, the model that was significant (*F*_(2, 29)_ = 10.773, *p* < 0.001) explained 38% (adjusted *R*^2^ × 100) of the state anxiety experienced by the children during quarantine. The pre-quarantine predictors that were significant were parent-child dysfunctional interaction (β = 0.405, *t* = 2.69, *p* < 0.05) and the marital status [1 = married or cohabitating; 0 = living alone] of their parents (β = −0.434, *t* = −2.94, *p* < 0.01).

In the case of parents, the model that was significant (*F*_(3, 28)_ = 17.170, *p* < 0.001) explained 61% (adjusted *R*^2^ × 100) of parental stress during quarantine. The pre-quarantine variables that were significant were the number of children (β = 0.256, *t* = 1.67, *p* < 0.05), marital status (β = −0.677, *t* = −4.82, *p* < 0.001), and the child’s behavior problems (β = 0.312, *t* = 2.07, *p* < 0.05).

### Educational Conditions and Impact of Quarantine on Children With Dyslexia, as Reported by Parents (RQ5)

Frequencies and percentages of the educational conditions and challenges are shown in Table 6. As shown, approximately 90% of parents reported having electronic devices for remote learning, although 71.9% also reported that such electronic devices were shared by other family members, such as siblings and parents. In relation to remote learning, 100% of teachers used online tasks through software such as Moodle, but only 21.9% occasionally attended their classes online with a teacher present. Parents reported that 56.2% of children spent less than 4 h per day on learning and 43.8% spent between 4 and 7 h. Further, 56.3% of children with dyslexia used audiobooks and/or readers for completing the school reading activities.

**TABLE 6:** Educational conditions and potential challenges reported by parents during Spain’s National Quarantine.

Summary of all survey questions and response options on the QQE	*N*	%
**Child has access to electronic devices to use for learning**		
Yes, and is the only person in household to use that device	6	18.8
Yes, but shared with others (siblings, parents)	23	71.9
No	3	9.4
**Child completes online classes with teachers**		
Yes	7	21.9
No	25	78.1
Online tasks (e.g., Moodle)	32	100
**The average time per day each child spends on learning**		
**(in total hours per day)**		
≤4 h	18	56.2
4–7 h	14	43.8
≥7 h	0	0
**Child needs help with homework during quarantine**		
Yes	32	100
No	0	0
**Average time per day supporting your child(ren) with learning**		
**during quarantine**		
Less than 1 h	0	0
1–2 h	3	9.4
3–4 h	23	71.9
More than 4 h	6	18.7
**Use of audiobooks and/or readers for school reading activities**		
Yes	18	56.3
No	14	43.8
**Difficulties in establishing study routines and with homework**		
Yes	32	100
No	0	0
**Difficulty completing tasks on time**		
Yes	20	62.5
No	12	37.5
**Quarantine has negatively affected your child’s learning**		
Yes	31	96.9
No	1	31.1
**Difficulties cooperating with the teacher**		
Yes	22	68.8
No	10	31.3
**The teacher helped me understand how to best support my child**		
**with homework**		
Yes	2	6.3
No	30	93.8
**Is special attention offered by your child’s teacher to improve reading skills**		
**during quarantine?**		
Yes, with similar amount of attention before quarantine	0	0
Yes, but with less attention during quarantine	25	78.1
No	7	21.9
**Which of the following are you most worried by during the quarantine**		
**(select all that apply):**		
The child’s learning and school success	32	100
Professional skills of child’s teacher	23	71.9
The child’s motivation and interest in learning and reading	27	84.4
The child’s peer relationships	25	78.1

*This survey was developed for the purposes of this study and to understand educational circumstances during Spain’s 2020 national quarantine and response to COVID-19. Each bolded item summarizes the question posed to the parent and immediately below each bolded item are the response options. QQE = Quarantine Questionnaire about Education.*

All parents of children with dyslexia reported difficulties in establishing study routines and homework completion. In fact, 100% of parents indicated their child needs help with homework. The majority of parents (90.6%) spent more than 3 h per day supporting their child’s learning and 96.9% (all but one parent) indicated that the pandemic has negatively affected their child’s learning. At the time of the survey, all but two parents responded “no” when asked if the child’s teacher assisted the parent with knowing how to best support the student with homework. Similarly, 68.9% of parents reported they had difficulties cooperating with the child’s teachers and that their child had difficulties completing tasks on time. The large majority (78.1%) of parents indicated that their child with dyslexia received less support for reading difficulties during quarantine, none received a similar amount, and 21.9% reported that the child received no special attention for their reading difficulties. Finally, the majority of parents were very worried about the child’s learning and school success (100%), the child’s motivation and interest in reading and in learning (84.4%), the child’s peer relations (78.1%), and the professional skills of the child’s teacher (71.9%).

## Discussion

The COVID-19 outbreak was a new and unexpected situation that quickly became a large-scale pandemic. Within Spain, and likely other countries, many have argued that the consequences of school closures and keeping children locked at home to prevent the spread of COVID-19 did not fully consider other important aspects of families’ and children’s wellbeing–including but not limited to their psychological and educational wellbeing (Smith, 2020; United Nations Educational, Scientific, and Cultural Organization [UNESCO], 2020). Recently published studies have begun to demonstrate several negative impacts of quarantine on students’ mental health and schooling (e.g., Brooks et al., 2020; Francisco et al., 2020; Gómez-Becerra et al., 2020; Orgilés et al., 2020, 2021), but to our knowledge, our study is the first to specifically examine the psychological and educational impact of the COVID-19 pandemic on students with dyslexia as well as the parents of students with dyslexia. It is also one of only a small number of studies with data collected about participants’ wellbeing shortly before and then again during the quarantine.

First and foremost, our findings highlight several negative psychological and educational impacts that national quarantine conditions can have on students with dyslexia and in their parents. Using self-report measures, our participants with dyslexia showed significantly increased symptoms of depression and state anxiety during the quarantine. This finding is generally consistent with other recent studies that included children and adolescents without learning disabilities (e.g., Cao et al., 2020; Duan et al., 2020; Francisco et al., 2020; Xie et al., 2020; Orgilés et al., 2021), but our study helps to evidence participants’ degree of mental health changes from before to during quarantine conditions. Our study also bolstered data from children’s self-reports about mental health by including parental reporting. Specifically, during the quarantine compared to before, we found that parents rated their child with dyslexia to have significantly more emotional difficulties, conduct problems, and hyperactivity-inattention (as measured with the SDQ). We also found that during the quarantine, the percentage of students with dyslexia who were above the clinical cut-off range was 53.1% for Emotional Symptoms, 21.9% for Conduct Problems, and 18.8% for Hyperactivity-Inattention. Our results are the first to document such changes from before to during quarantine conditions, but children’s presence of difficulty during the quarantine correspond well with the findings reported by Gómez-Becerra et al. (2020). In their study, which evaluated a wide range of individuals during the quarantine between 3 and 18 years old (all living in Spain), they found similar percentages of students with clinically elevated scores on the SDQ: 48.6% for Emotional Symptoms, 24.2% for Conduct Problems, and 20.3% for Hyperactivity-Inattention.

Conditions of COVID-19 and Spain’s national quarantine also had a negative influence on our participants’ reading motivation and reading activity. For instance, during quarantine, children reported significantly less motivation for reading on measures of the MRP and TVS-C and significantly less breadth of reading on the RAI. These findings are important because they clearly highlight a critical need to support students with dyslexia during atypical schooling conditions, just as it is well understood that such students should be well supported under more typical school conditions (Nelson and Harwood, 2011a; Morte-Soriano et al., 2020). These findings are also important because one cannot assume that most students will have less reading motivation during quarantine conditions. For instance, studies of adults and children without learning disabilities suggest that many such individuals have spent even more time reading, and read with greater breadth, during quarantine conditions (Adeyemi, 2020; Tyagi et al., 2020).

Another goal of our study was to explore the predictive power of different pre-quarantine variables to potentially explain emotional problems in children with dyslexia during quarantine. Thus, pre-existing conditions like difficult interaction between parent-child, and living with a divorced / separated or single parent, seem to have a great impact on the state anxiety of children with dyslexia. Previous research has shown that parental status (e.g., divorced or single) seems to comprise a risk factor for fear and anxiety, and health risks and fear connected to COVID-19 influence parents’ levels of stress both at the individual level (e.g., being over-reactive, feeling nervous, irritable) and at the dyadic level (e.g., parent-child dysfunctional interactions, finding it difficult to enjoy interactions with the child, and child behavioral and emotional expressions); and, as a consequence, children’s psychological problems are impacted (Spinelli et al., 2020). Similarly, Orgilés et al. (2020, 2021) found a relationship between parents’ stress and their children’s emotional state. In other words, these studies show that anxiety and depression symptoms were more likely in children whose parents reported a higher level of stress. In fact, primary caregivers’ level of stress was related to 25 of 31 child symptoms (Orgilés et al., 2020). As Marchetti et al. (2020b) point out, parents experiencing high psychological distress may be less attentive to and warm with their children, and they may also transfer the burden of their emotional distress to their children, which could affect their children’s adjustment.

Our study also highlighted the impacts of quarantine conditions and the relative wellbeing of parents who have a child with dyslexia. Findings showed that compared to pre-quarantine conditions, mothers reported significantly greater stress and undesired interactions with their child during quarantine. These findings are new and important to report in the context of evaluating parents of students with dyslexia, but the data appear consistent with what might be expected during a quarantine. For instance, some studies point out that dealing with confinement is a particularly stressful experience for parents who must balance personal life, work, and raising children (Marchetti et al., 2020a,b; Mazza et al., 2020a,b; Spinelli et al., 2020). In the case of students with dyslexia, parents must cope with poor academic progress, a greater need to help with homework, and seek appropriate help for reading instruction or support. Additionally, approximately one-third of mothers scored above the clinically significant range on the GHQ-12, further elucidating the high levels of psychological distress (an emotional state characterized by depressive and anxious symptoms) during quarantine conditions. These results are generally consistent with those reported by others. In Spain, Rodríguez-Rey et al. (2020) reported that 37% of adults experienced psychological distress, in China Liang et al. (2020) reported that 40.4% of youth were prone to psychological distress and in Italy, more than 80% of their sample reported high levels of psychological distress and 17% reported parenting-related exhaustion (Marchetti et al., 2020b).

The present results also revealed some predictors of parents’ stress during quarantine, such as number of children, marital status (e.g., divorced or single), and the child’s behavior problems. This finding is in line with previous studies (Mikolajczak et al., 2018; Marchetti et al., 2020a,b; Spinelli et al., 2020) that have highlighted risk factors for parenting stress, relating to socio-demographic characteristics (e.g., having a larger number of children, lacking a domestic partner) or child features (e.g., special needs, externalizing symptoms, ADHD, behavior problems). However, further studies should examine this issue in even greater depth.

Consistent with parental reports of distress, most if not all the parents in our study reported, for example (a) their child having difficulty with establishing study routines and completing school-related tasks on time, (b) spending on average three or more hours each day assisting children with learning, (c) negative overall impacts on their child’s learning and less attention from the child’s teacher to support reading skills, and (d) not getting sufficient support from the child’s teacher. Obviously, facing such circumstances during more than a month of quarantine is likely to significantly increase parents’ risk of stress, including parents of children without learning disabilities (Spinelli et al., 2020).

### Study Limitations, Implications, and Future Research Directions

As an initial evaluation of quarantine conditions on students with dyslexia and their parents, our study should be understood while considering its limitations. For example, this study used child and parent wellness reports; however, only mothers completed all measures. Mothers’ and fathers’ ratings should be considered in future studies in an attempt to determine whether mothers or fathers perceive wellbeing similarly. Also, future research should attempt to add observational measures. This is indeed a challenging task during quarantine, but with significant resources, video recording could be used as a possible means of direct observation. Also, our sample is not representative of all students in Spain (or students globally) with dyslexia and future research is needed over time to gather data from a larger and even more representative sample. Similarly, future research should aim to broaden our understanding of how quarantine conditions impact children’s educational and psychological wellbeing by including additional measures (e.g., direct assessments of reading during quarantine, more comprehensive assessments of depression or anxiety) and exploring possible mediators or moderators among variables. Components of time (e.g., for how long has the child or parent been experiencing quarantine) and specific behavior (e.g., to what degree has “typical” educational conditions, such as in-person schooling, changed during quarantine) must also be examined to understand short-term and long-term impacts of quarantine. Although it is impossible to know for sure right now whether negative impacts of quarantine will improve or worsen over time, many have argued that psychological and educational impacts will worsen over time (Brooks et al., 2020; Gómez-Becerra et al., 2020), especially for those who are most economically, educationally, or psychologically vulnerable (Miranda et al., 2005, 2008, 2013; Soriano-Ferrer and Contreras, 2012; Bonifacci et al., 2019; Asbury et al., 2020; Colizzi et al., 2020; Marchetti et al., 2020a; Smith, 2020; Iriarte-Redín et al., 2021).

With limited research currently available on the impacts of quarantine conditions on children with dyslexia and their parents, we choose not to overstate our findings by offering in depth implications or recommendations. One simple observation is that much more research is needed to better understand the impacts of COVID-19 on educational and psychological wellbeing and understand how to best address all negative impacts with prevention and intervention. Clearly, negative impacts were observed during quarantine compared to pre-quarantine conditions for the participants in our study (and those of other studies), but given the unprecedented circumstances of this pandemic, it is unknown to what degree we would expect this level of negative impact. A pandemic is inevitably going to have negative impacts across a range of areas–including but not limited to mental health and education–but it is impossible to know at this time to what extent national policies and practices during quarantine minimized the greatest possible harm of a pandemic. For this reason, national and international research directly examining psychoeducational impacts of COVID-19 (and quarantines more specifically) is essential so that policies and strategies can align with needs and actions that best minimize harm in large-scale and equitable ways. Using frameworks of internationalization (e.g., van de Vijver, 2013; Begeny, 2018; Begeny et al., 2018; Bernardo et al., 2018), such research (within and across countries) can leverage understanding, knowledge, and culturally appropriate best practices for schooling, mental health, and more. Although COVID-19 is a global concern, its impacts may look differently within and across different countries; thus, an internationalization framework for research can help researchers, service providers, and policy makers to understand, for example, how different countries are attempting to evaluate and address psychological and educational impacts–and to what extent can we learn from other nations’ efforts to minimize harmful effects of mandatory quarantine on psychoeducational outcomes.

Until more abundant research is available, converging evidence in the past year offers one clear implication: because quarantine conditions are negatively impacting large percentages of children and families, immediate and evidence-informed actions must be used to remediate or at least slow the widespread, negative, psychoeducational impacts. In numerous ways, psychologists and educators can help in this effort, including efforts to assist students with dyslexia and their families. For example, professionals can work to implement virtual interventions if face-to-face intervention is not possible or feasible–and in doing so, aim to select interventions that have contextually appropriate evidence of being effective (at least within face-to-face contexts if virtual implementation has not yet been tested) and closely attend to intervention effectiveness at the individual level so that treatment adjustments can be made immediately to best support the individual. Intervention or prevention efforts must also consider multiple aspects of students’ learning or psychological wellbeing, such as by offering guidance for students’ teachers within schools and, when appropriate, for parents in home. As evidenced in numerous studies well before COVID-19 (e.g., Soriano-Ferrer et al., 2011), intervention may need to be individualized or intensified for students with dyslexia.

Indeed, a rapid and focused effort on prevention, intervention, and careful evaluation of intervention effectiveness is greatly needed at an unprecedented time that currently lacks the needed amount of research-informed guidance. Although “basic” needs (e.g., food, water, safety from the coronavirus, suitable healthcare) are essential during a time of crisis, we argue that psychoeducational needs are equally essential and that local schools, national governments, and global agencies must do even more to provide needed and equitable supports. These supports are even more critical to students with learning difficulties and those who do not have access to equitable educational conditions (e.g., those limited by economic disadvantages).

## Data Availability Statement

The raw data supporting the conclusions of this article will be made available by the authors, without undue reservation.

## Ethics Statement

The studies involving human participants were reviewed and approved by University of Valencia. Written informed consent to participate in this study was provided by the participants’ legal guardian/next of kin.

## Author Contributions

MS-F have contributed to the conception and design of work, to interpretation of data, drafting the manuscript and revising it critically for important intellectual content, a final approval of the version to be published, and to agreement to be accountable for all aspects of the work in ensuring that questions related to the accuracy or integrity of any part of the work are appropriately investigated and resolved. JB have contributed to analysis and interpretation of data, drafting the manuscript or revising it critically for important intellectual content, a final approval of the version to be published, and to agreement to be accountable for all aspects of the work in ensuring that questions related to the accuracy or integrity of any part of the work are appropriately investigated and resolved. MM-S and EP-M have contributed to acquisition of data, to interpretation of data, drafting the manuscript or revising it critically for important intellectual content, a final approval of the version to be published, and to agreement to be accountable for all aspects of the work in ensuring that questions related to the accuracy or integrity of any part of the work are appropriately investigated and resolved. All authors contributed to the article and approved the submitted version.

## Conflict of Interest

The authors declare that the research was conducted in the absence of any commercial or financial relationships that could be construed as a potential conflict of interest.
